# Detecting Airway Involvement in Non-Asthmatic Eosinophilic Disorders: Diagnostic Utility of Fractional Exhaled Nitric Oxide (FeNO)

**DOI:** 10.3390/arm93050036

**Published:** 2025-09-16

**Authors:** Nicolas Raoul, Lucie Laurent, Ophélie Ritter, Pauline Roux-Claudé, Faraj Al Freijat, Nadine Magy-Bertrand, Virginie Westeel, Cindy Barnig

**Affiliations:** 1 Department of Chest Disease, University Hospital Besançon, F-25000 Besançon, France; 2 Department of Chest Disease, Hôpital Nord Franche Comté, F-90400 Trévenans, France; 3 Division of Internal Medicine, CHU-Besancon, F-25000 Besancon, France; 4 Université Marie et Louis Pasteur, EFS, INSERM, UMR RIGHT, F-25000 Besancon, France; 5 INSERM, F-CRIN, Clinical Research Initiative in Severe Asthma: A Lever for Innovation & Science (CRISALIS), France

**Keywords:** hypereosinophilia, FeNO, HES

## Abstract

**Highlights:**

**What are the main findings?**

- In patients with persistent eosinophilia from disorders other than asthma, elevated FeNO levels were associated with airflow obstruction and respiratory symptoms such as cough and wheezing.
- Unlike in asthma, blood eosinophil counts showed no correlation with FeNO levels.

**What is the implication of the main finding?**

- FeNO measurement can help detect bronchial involvement in non-asthmatic eosinophilic disorders where respiratory symptoms may be less apparent or masked by extra-respiratory manifestations.
- Combining FeNO assessment with clinical evaluation could facilitate earlier and adapted treatment for patients with eosinophilic bronchial involvement who might otherwise go unrecognized.

**Abstract:**

Airway involvement in eosinophilic disorders other than asthma is not well-defined, and the symptoms may be overshadowed by other more prominent eosinophilic extra-respiratory manifestations. This study aimed to evaluate the utility of fractional exhaled nitric oxide (FeNO) in diagnosing eosinophilic airway involvement in patients with persistent eosinophilia (>0.5 × 10^9^/L). We conducted a retrospective analysis of adult patients with confirmed peripheral blood eosinophilia (>0.5 × 10^9^/L) on at least two occasions one month apart. Patients with blood eosinophilia associated with known eosinophilic airway inflammatory diseases were excluded from the study. Pulmonary function testing, spirometry, and FeNO measurement were conducted. A total of 14 patients with various eosinophil-related disorders were identified, with a mean age of 65.7 years. Increased FeNO levels were associated with airflow obstruction and clinical symptoms such as coughing and wheezing. Notably, eosinophil levels were not predictive of eosinophilic airway involvement. FeNO could be a useful diagnostic tool for detecting bronchial eosinophilic airway inflammation in non-asthmatic disorders, thereby enabling appropriate treatment. Further studies with larger cohorts are needed to validate these findings.

## 1. Introduction

Eosinophilic granulocytes, a subset of leukocytes, are typically present in low numbers in the bloodstream, constituting less than 5% (<0.5 × 10^9^/L) of the leukocyte population [1]. Originating from the bone marrow, these cells undergo transient circulation in the blood before homing to various tissues. Under normal homeostatic conditions, eosinophils are recruited to different tissues such as the digestive system, adipose tissue, mammary glands, thymus, and uterus, although their precise functions in these tissues are not fully understood [1].

Numerous disorders are characterized by elevated eosinophil levels in the bloodstream with consequent excessive accumulation in tissues, resulting in tissue damage and organ dysfunction [2]. Eosinophils can accumulate in the airways leading to bronchial inflammation and bronchoconstriction, particularly evident in asthma, whether associated with allergies, nasal polyps, allergic bronchopulmonary aspergillosis, or eosinophilic granulomatosis with polyangiitis (EGPA) [3]. Eosinophilic airway accumulation can also be observed during eosinophilic pneumonia, where involvement however predominantly locates to the lung parenchyma [4]. Outside of this context, there is limited knowledge about the involvement of airways in other hypereosinophilic conditions, including in blood eosinophilia of undefined reason that is considered as asymptomatic.

Fractional exhaled nitric oxide (FeNO) is a well-established non-invasive method for measuring eosinophilic airway inflammation [5]. Due to its various properties, simplicity, and speed of handling as a complementary examination, FeNO is utilized as an aid in the diagnosis of asthma, as a marker of severity, and as a predictor of response to treatment, particularly inhaled corticosteroids in asthma [6].

In this study, our objective was to evaluate the diagnostic utility of FeNO in detecting eosinophilic airway involvement in patients with persistent eosinophilia (>0.5 × 10^9^/L), who do not present with conditions typically associated with eosinophilic airway inflammation, such as asthma.

## 2. Materials and Methods

For this, we conducted a retrospective analysis of adult patients (>18 years) with peripheral blood eosinophilia (>0.5 × 10^9^/L), confirmed at least on two occasions one month apart and who underwent pulmonary function testing including spirometry, bronchodilator responsiveness testing, and FeNO measurement at the Respiratory Physiology Department of Besançon University Hospital as well at the Respiratory Department of Hôpital Nord Franche Comté, France between January 2022 and February 2024. This study was approved by the Institutional Review Board of the French Society of Respiratory Medicine (CEPRO 2024-014).

Routine spirometry (Platinum Elite, MGC Diagnostics Corporation, Saint Paul, MN, USA) was performed in all patients in accordance with current standards [7]. Predicted normal values were derived from the Global Lung Function Initiative (GLI) reference equations, in line with current recommendations [8]. FeNO was measured using the Medisoft device (Sorinnes, Dinant, Belgium) in accordance with ATS/ERS recommendations [5]. A FeNO value ≥25 parts per billion (ppb) was considered as indicative of significant bronchial inflammation [5].

Patients with blood eosinophilia associated with known eosinophilic airway inflammatory diseases (asthma, nasal polyposis, allergic bronchopulmonary aspergillosis, EGPA and eosinophilic pneumonia) were excluded from the study.

The most recent blood eosinophil level available within a 15-day timeframe was considered for analysis.

Median values of various variables were compared by a Mann–Whitney test. Additionally, we utilized linear regression analysis. Data are presented as means (±standard deviation; SD), medians (interquartile ranges; IQR) or number of patients (%). A significance threshold of *p* < 0.05 was applied.

## 3. Results

A total of 14 patients were identified (seven women, seven men), with a mean age of 65.7 years (±15.5) (range, 21 to 85 years) (Table 1). The causes of hypereosinophilia varied. Among the included patients, three had idiopathic hypereosinophilic syndrome, one had hyper-IgG4 related disease, one had a solid organ malignancy, and one had eosinophilic fasciitis, Eight cases remained of unidentified etiology, despite extensive evaluation, including systematic exclusion of parasitic infections, atopic dermatitis, and drug-induced eosinophilia, and were therefore classified as idiopathic hypereosinophilia. As specified in the Methods section, patients with blood eosinophilia associated with known eosinophilic airway inflammatory diseases (asthma, nasal polyposis, allergic bronchopulmonary aspergillosis, eosinophilic granulomatosis with polyangiitis, and eosinophilic pneumonia) were excluded from the study.

Medical records revealed that patients frequently reported respiratory symptoms. Dyspnea (mMRC ≥ 1) was the most common, affecting 57.1% of patients, followed by cough (42.9%) and wheezing (28.6%). A history of tobacco use was present in eight patients (57%), with two (14%) continuing to smoke at the time of evaluation. Chest computed tomography (CT) scans, available for 11 patients, showed emphysema in one case and mild ground-glass opacities in two cases.

**Figure 1:**
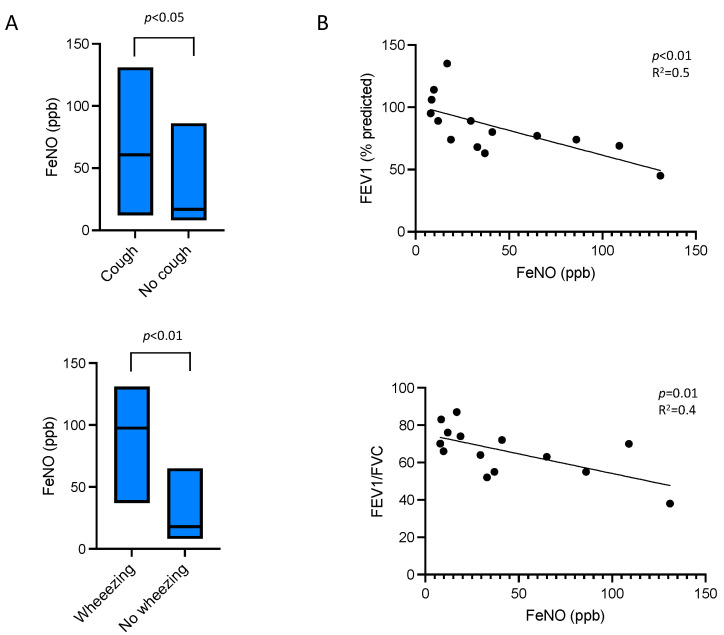
FeNO levels in non-asthmatic eosinophilic disorders. (**A**) Median analysis (Floating bars, line at median, min to max) and (**B**) correlation with lung function parameters.

Regarding FeNO levels, eight patients (57.1%) exhibited increased values. Patients experiencing cough had significantly higher FeNO levels in contrast to those without cough (41.0 ppb (IQR: 29.5–109) vs. 16.9 ppb (IQR: 8.5–33) (*p* ≤ 0.05)) (Figure 1, upper panel A). Similarly, for individuals reporting wheezing, FeNO levels were also significantly higher (97.5 ppb (IQR: 49.25–125.5) vs. 17.9 ppb (IQR: 9.47–35) (*p* ≤ 0.01)) (Figure 1, lower panel A). There was also a tendency for higher FeNO levels in patients with dyspnea (37 ppb (IQR: 20.75–97.50) vs. 16.9 ppb (IQR: 8.25–29.95) (*p* = 0.06)).

Pulmonary function tests revealed a mean forced expiratory volume in 1 s (FEV1) of 84.1% (±23.0) and a mean forced vital capacity (FVC) of 100.1% (±16.8) of the predicted values. Notably, eight patients (57.1%) exhibited significant airflow obstruction, with a significant bronchodilator response in three patients (21.4%). Furthermore, a significant relationship was observed between FeNO levels and FEV1 values (*p* < 0.01, R^2^ = 0.5), as well as the FEV1/FVC ratio (*p* = 0.01, R^2^ = 0.4) (Figure 1B). Interestingly, blood eosinophil levels were not predictive of eosinophilic airway involvement, as they showed no correlation with FeNO levels (r = 0.1, *p* = 0.2).

## 4. Discussion

Unlike asthma, which only involves the airways, various eosinophil-related disorders can affect multiple organ systems, including the airways too [4]. However, the prevalence of eosinophilic airway involvement beyond asthma is not well-defined in eosinophilic disorders, as a result of absence of consensus regarding documentation and diagnosis. Moreover, symptoms related to eosinophilic airway involvement are often overlooked, or may be overshadowed by other, more prominent extra-respiratory manifestations. In cases of idiopathic Hypereosinophilic Syndrome (HES), lung involvement is frequently observed, with a variety of radiological findings in the pulmonary parenchyma identified in at least 25% of the patient population [9]. However, the incidence of airway involvement in HES is reported to be relatively rare [10]. Despite its infrequency, there are documented case reports illustrating patients with HES exhibiting asthma-like symptoms and where eosinophilic infiltration of the airways has been confirmed through detailed bronchial histopathological analysis [11,12].

Despite the small sample size and heterogeneity within our patient cohort, our study unveils significant insights. Most notably, we observed a significant correlation between elevated FeNO levels and airflow obstruction, as well an association with respiratory symptoms indicative of bronchial involvement, such as coughing and wheezing.

In asthma, FeNO is consistently elevated in patients with eosinophilic airway inflammation, as supported by extensive literature. FeNO has demonstrated high specificity for confirming the diagnosis in asthma and retains diagnostic value even in the absence of spirometric reversibility [13]. Moreover, in asthma, FeNO levels correlate with cough, wheeze, and variable airflow obstruction [6].

The significance of FeNO extends to non-asthmatic airway diseases. In chronic cough, it helps identify cough-variant asthma [14,15], supports the diagnosis of non-asthmatic eosinophilic bronchitis [14,15], and predicts corticosteroid responsiveness [16]. Notably, FeNO elevation may occur even without wheeze, underscoring its ability to detect bronchial involvement beyond classic features. This aligns with our study, where FeNO proved valuable in revealing bronchial involvement even in the absence of typical manifestations.

COPD is not classically considered an eosinophilic disease, yet eosinophilic inflammation can be encountered in a subset of patients [17]. In this context, FeNO may also provide useful information; although average values are generally lower and more variable than in asthma, some studies report that persistently elevated FeNO (>20 ppb) predicts exacerbation risk [17,18] and helps identify an eosinophilic phenotype responsive to inhaled or systemic corticosteroids [19,20].

Interestingly, our findings indicate an absence of correlation between blood eosinophil levels and FeNO measurements, diverging from the pattern observed in asthma, where a strong positive correlation is well-established [21]. In asthma, this relationship reflects converging type 2 pathways within the bronchial mucosa, driven by allergens and other environmental stimuli: local IL-4/IL-13 production induces iNOS and FeNO release, while IL-5 regulates eosinophil recruitment, maturation, and survival [22]. By contrast, in eosinophilic disorders such as HES, airway involvement is not systematic; even in the presence of marked blood eosinophilia, eosinophils may be recruited to other organs rather than the bronchi. The distribution of tissue involvement is highly unpredictable, which explains the lack of correlation observed in our study. In this context, FeNO provides specific added value by helping to identify bronchial involvement beyond what blood eosinophil counts alone can reveal.

This study has several limitations. First, its retrospective design and small sample size limit the generalizability of the findings. Second, the heterogeneity of eosinophilic disorders adds complexity, as airway involvement may differ across conditions Although all patients with elevated FeNO and airway obstruction were proposed anti-asthmatic treatment following positive screening, no systematic follow-up was available to evaluate the treatment responsiveness that might have been expected. Further prospective studies are needed to validate and extend these observations.

## 5. Conclusions

In conclusion, our results highlight the importance of FeNO as a diagnostic marker for detecting eosinophilic airway inflammation in individuals with persistent eosinophilia due to disorders other than asthma. These findings suggest that combining FeNO measurements with clinical evaluations can improve recognition of bronchial involvement in eosinophilic disorders that might otherwise be overlooked, thereby enabling timely and appropriate treatment. Further research with larger cohorts is needed to confirm these insights.

## Figures and Tables

**Table 1:** Demographic and clinical characteristics of the study subjects.

	*n* = 14
Age (years)	65.7 ± 15.5
Gender (M/F)	7/7
BMI (kg/m^2^)	24.7 ± 4.0
Blood eosinophil count (×10^9^/L)	1.45 (0.9–3.1)
Causes of hypereosinophilia	
Idiopathic HES	3 (21.4%)
Ig4-related disease	1 (7.1%)
Eosinophilic fasciitis	1 (7.1%)
Solid malignant tumor	1 (7.1%)
Idiopathic hypereosinophilia	8 (57.1%)
Respiratory symptoms	
Cough	6 (42.9%)
Dyspnea (≥1 mMRC)	8 (57.1%)
Wheezing	4 (28.6%)
FeNO (ppb)	31.25 (11.45–70.25)
Pulmonary function tests	
FEV1 (L)	2.07 ± 0.75
FEV1 (% predicted)	84.1 ± 23.0
FVC (L)	3.10 ± 0.99
FVC (% predicted)	100.1 ± 16.8
FEV1/FVC (%)	66.1 ± 13.0

Data are presented as means ± SD, medians (interquartile ranges) or number of patients (%). Abbreviations: BMI = Body Mass Index; FeNO, Fractional Exhaled Nitric Oxide; FEV1, forced expiratory volume in 1 s; FVC, forced vital capacity; HES, Hypereosinophilic syndrome, mMRC, modified Medical Research Council.

## Data Availability

The raw data supporting the conclusions of this article will be made available by the authors on request.

## Abbreviations

The following abbreviations are used in this manuscript:

FeNO	Fractional Exhaled Nitric Oxide
FEV1	Forced Expiratory Volume in 1 Second
FVC	Forced Vital Capacity
HES	Hypereosinophilic Syndrome
mMRC	Modified Medical Research Council
